# Obituary Notice

**Published:** 1855-02

**Authors:** 


*Obituary Notice*—

At a meeting of the students of Rush Medical College, held January 25th, 1855, L. H. Kennedy, M. Davis, James L. Suddeth, John McHugh, and W. B. Cooper, who had been previously appointed a committee to draft suitable resolutions relative to the death of M. A. Warson, and G. C. Hughes, reported the following preamble and resolutions, which were unanimously adopted:

Whereas it has pleased Almighty God, in His wise Providence, to remove from our midst, our worthy and highly esteemed friends and class-mates, M. A. Warson, and G. C. Hughes. Therefore,

*Resolved*, That we deeply feel the loss of our deceased class mates who. by their upright moral conduct, and gentlemanly deportment, had endeared them to all with whom they were associated.

*Resolved*, That in their death, this class is deprived of two worthy members, the profession of devoted students, who, had life been prolonged, bid fair to become honorable members of the profession they had chosen.

*Resolved*, That we deeply sympathize with their relatives in their bereavement, and while we mourn their loss, may we be reminded that we, too, are travelling to that "land of deepest shade unpierced by human thought."

*Resolved*, That the thanks of this class be tendered to their room mates, for the untiring attention they bestowed during the affliction of our deceased friends.

*Resolved*, That we hereby tender our thanks to Professors Davis and Freer, for their constant attention during the illness of our deceased class-mates, and deep solicitude for their welfare, also for their presence and kind assistance at their funeral.

*Resolved*, That these resolutions be published in the North Western Medical and Surgical *Journal*, and the Chicago Daily *Journal*, and that a copy of the same be sent to their relatives.

C. GOODBRAKE, Chairman.

R. S. HITT, Sec.

*To Readers.*—The valuable contribution of Dr. Williams, and the Medical testimony in the case of Green, filled so much more space than we expected, that several articles are crowded out.

